# Utilizing shallow seismic reflection for mitigating seismic and geo-environmental hazards in the development project of Gabal Ataqa Area, Cairo–Suez District, Egypt

**DOI:** 10.1038/s41598-023-43904-2

**Published:** 2023-10-07

**Authors:** Hossameldin M. Mohammad, Sherif M. Elhady, Adel Kotb, Mohammad Ezzelarb, Alhussein Adham Basheer

**Affiliations:** 1 National Research Institute of Astronomy and Geophysics, (NRIAG), Helwan, Cairo 11421 Egypt; 2 Geology Department, Faculty of Science, Helwan University, Ain Helwan, Helwan, Cairo 11795 Egypt

**Keywords:** Geophysics, Seismology, Tectonics

## Abstract

Nowadays, development projects are becoming so rapid in many developing countries worldwide. The study of interest focuses on Cairo–Suez District, which represents the most important location in Egypt for major infrastructure projects and urbanization expansion. Consequently, it is very important to reduce the hazards surrounding this area from natural disasters, so all information that supports geo-environmental hazards assessment is importantly needed. The Gabal Ataqa area has socioeconomic importance as it is considered the industrial zone in the national project for developing the northern part of the Gulf of Suez. The situation of Gabal Ataqa area for being rich with numerous geological structures, and its socioeconomic importance calls the need for the delineation of the subsurface structural features in this area using the appropriate method. The geological framework has recognized all Quaternary faults in the Gabal Ataqa area as they are exposed to the surface, at the same time, no seismic reflection data has been acquired in the Gabal Ataqa area. Accordingly, the shallow seismic reflection method is applied in the Gabal Ataqa area to detect the Quaternary faults that are not exposed to the surface and also to detect any subsurface features that may cause construction problems, such as water-wet sands, sabkhas, and limestone cavities. Three lines of 2D seismic profiles are acquired in the study area. After applying seismic data processing and interpretation, two Quaternary faults have been recognized in the first and third lines. The location of the first seismic line has been chosen to be between two historic earthquake events, increasing the probability that the sources causing these two events will be located on the newly recognized quaternary fault. A dim spot has been recognized in the second seismic line at a depth of 50 m, which may indicate the presence of a groundwater aquifer or wet sandstone layer. Based on these results, changing the industrial zone place to another side to be away from the area of the three seismic lines is highly recommended.

## Introduction

**Figure 1:**
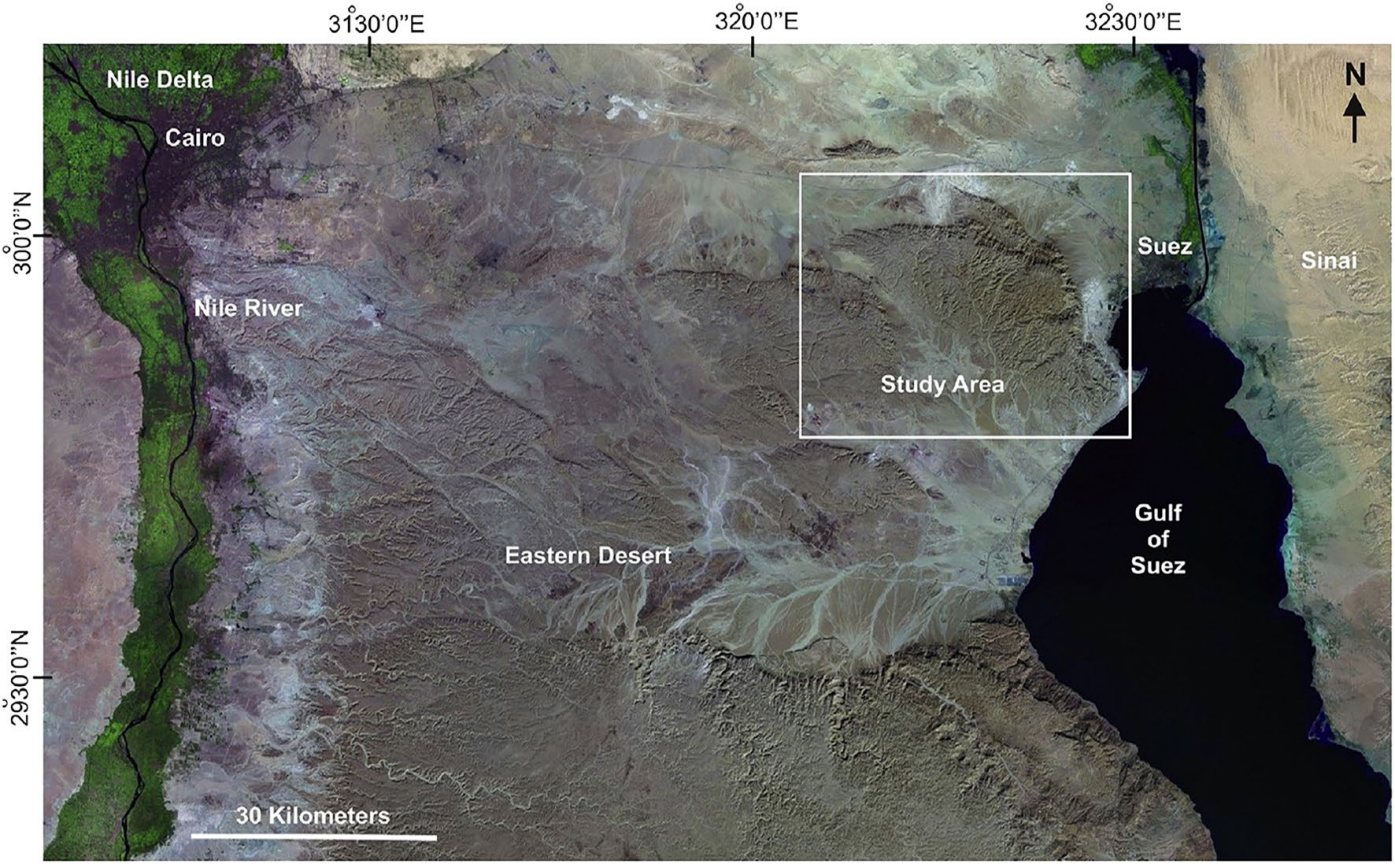
Location of the study area.

Several international seismological surveys indicate that active faults are considered the main reason for earthquakes^1^; therefore, recognizing and identifying the location of an active fault is very important and highly needed^2^. The Cairo–Suez District is a portion of the unstable shelf units that represent the majority of northern Egypt, where the current study is located (Fig. 1). Because of the tectonic disruption caused by this mobile shelf, structural highs and lows can be clearly seen (e.g., Gabal Ataqa, Abu Treifiya, Gabal El-Qattamiya, Gabal Nasuri, Gabal Abu Shama, and Gabal Mokattam).

**Figure 2:**
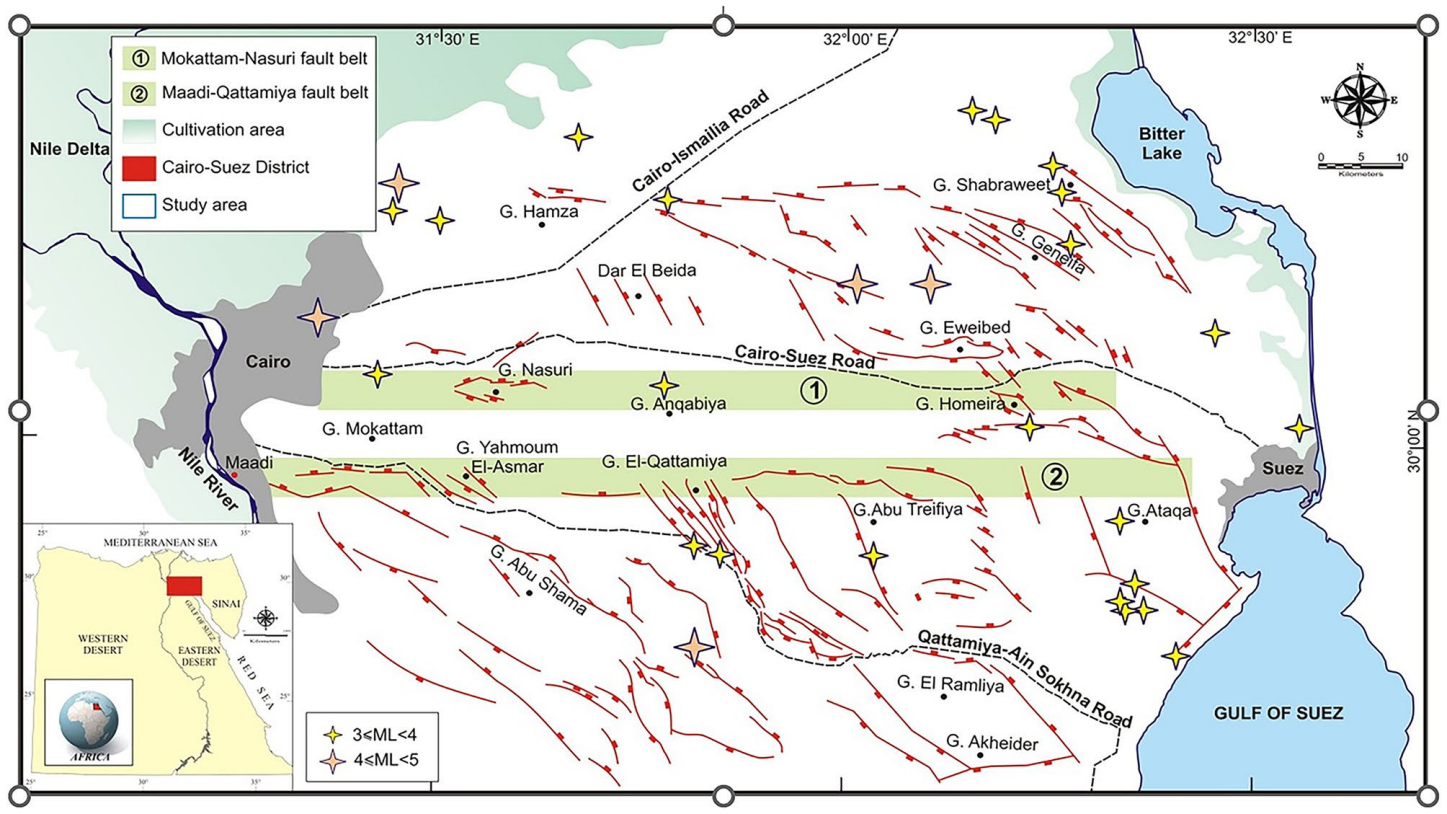
Simplified tectonic map of Cairo-Suez District and earthquake epicenters marked with stars (Hussein et al., 2013).

Based on an amalgamation of information from the National Earthquake Information Center (NEIC), the International Seismological Center (ISC), and available data in research papers^3^, the Cairo–Suez District contains a zone of low to moderate seismicity, as shown in Fig. 2^4^. Moreover, the Cairo–Suez District has normal faults with a secondary strike-slip component, which could be extremely important in the assessment of seismic hazards.

The geological framework interpreted all exposed quaternary faults in the Gabal Ataqa area. Still, the same framework could not detect the other quaternary faults as they were not exposed to the surface, increasing the need for another method to detect them. Some of these quaternary faults might be classified as active faults, increasing the earthquake's probable damage.

Since the 90th seismic reflection method has become a primary tool for detecting defects^5^. The subsurface image obtained from seismic data helps in the determination of the depth and thickness of the strata, as well as lateral variations and tectonic disturbances^6^. As a result, lately, there has been extensive utilization of the shallow seismic reflection technique to map underground characteristics for engineering purposes. Detecting a rise in groundwater levels has the potential to reduce the bearing capacity of the underlying rock. This reduction in bearing capacity can, in turn, impact the stability of the ground^7,8^. In addition, applying the seismic reflection method provides significant information about the individual faults in the area, this information is highly needed for seismotectonic studies.

**Figure 3:**
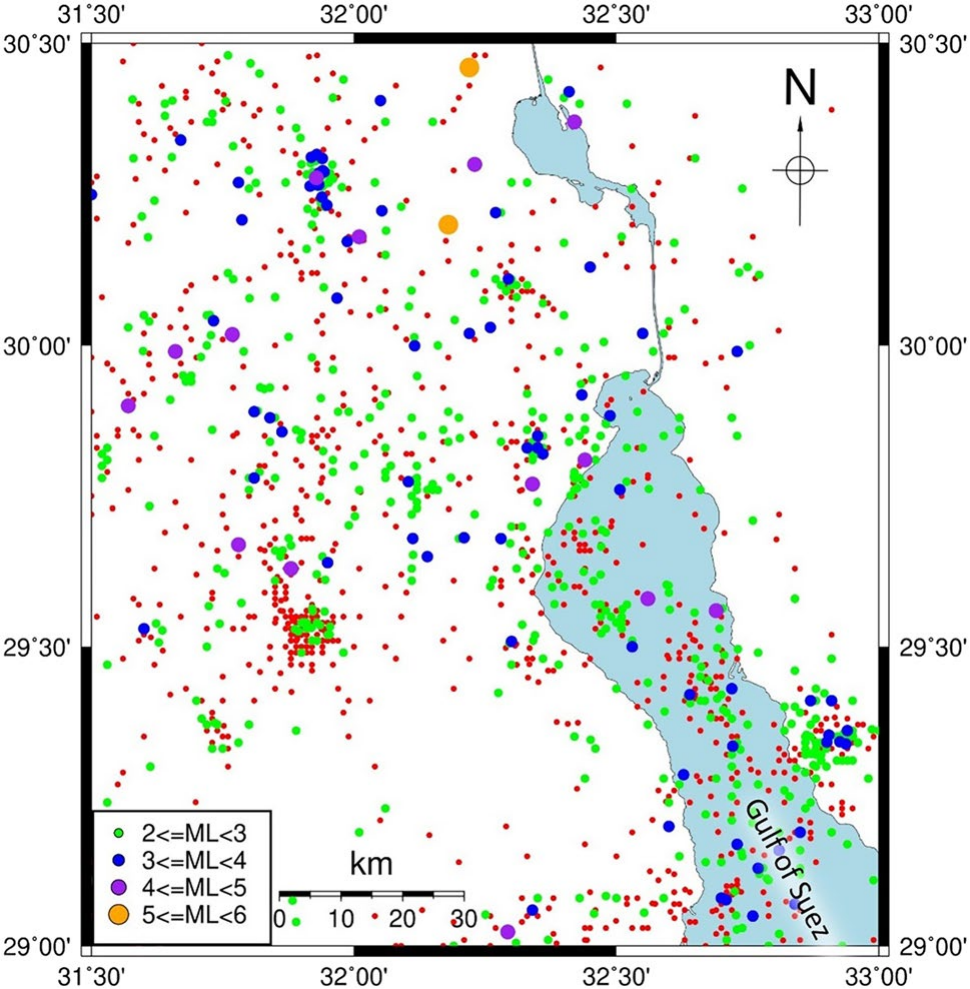
Seismicity of the northwestern edge of the Gulf of Suez.

Seismotectonic involves the examination of the interconnections among earthquakes, active tectonic processes, and specific faults within a particular geographical area. It seeks to understand which faults are responsible for seismic activity in a certain region^9^. Accordingly, providing such information about the shallow subsurface structure is very important to assess the earthquake hazard and minimize the probable earthquake damage^10^. Due to the progress in computational methods, the repercussions of an earthquake can be anticipated on any given development zone^11,12^. By analyzing the historical earthquake data obtained from the Egyptian National Seismological Network (ENSN), over the span of ten years, starting in 2009 and ending in 2019^13^, the northwestern edge of the Gulf of Suez was affected by low to moderate seismic activity (Fig. 3).

**Figure 4:**
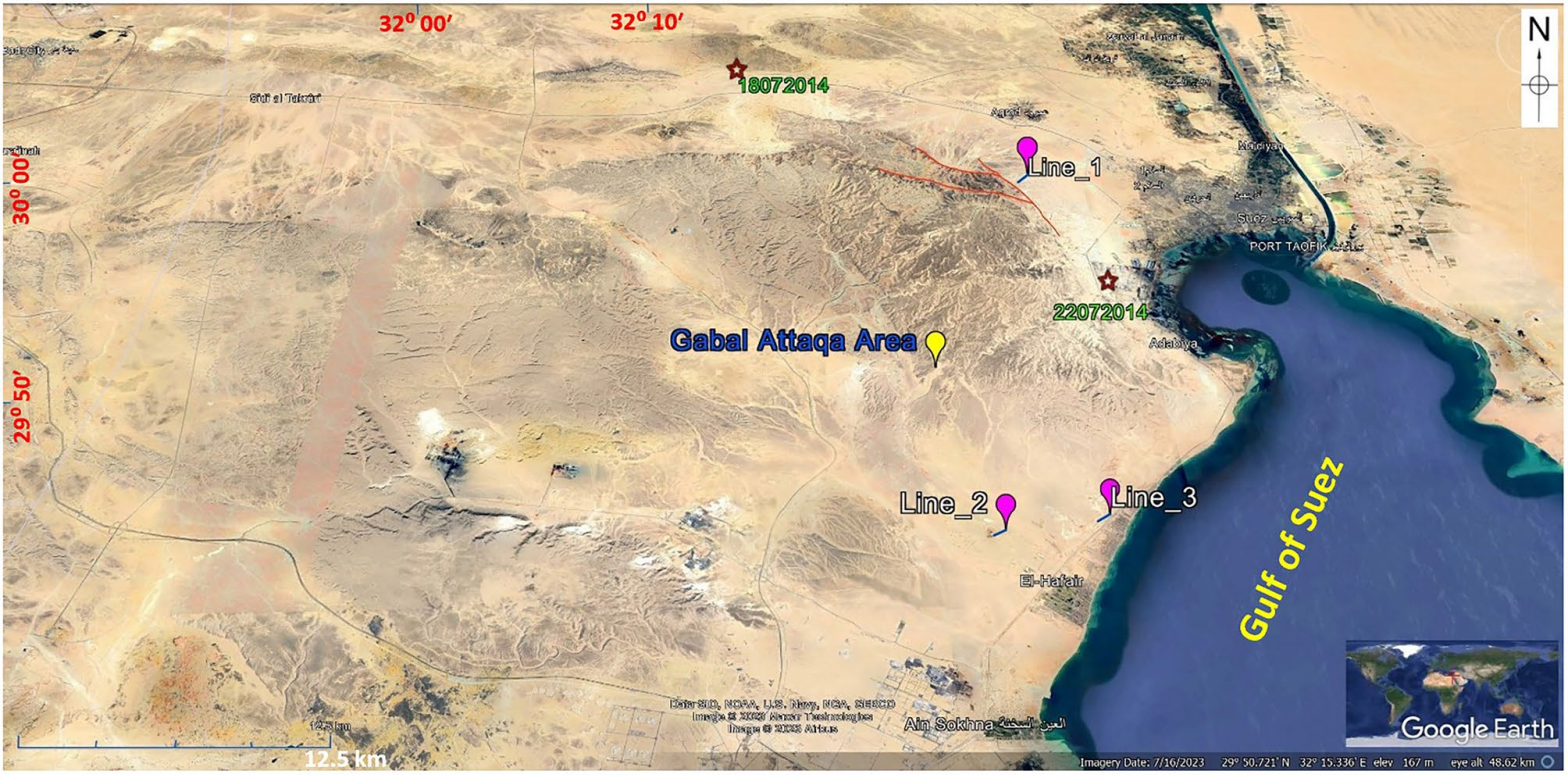
Three measured seismic lines (Line_1, Line_2 and Line 3), plotted on Google Maps, the red lines represent the exposed Quaternary faults (interpreted by the geological framework).

The major fault directions in the Gabal Ataqa area are NW–SE-oriented faults. So, three 2D seismic lines are acquired in the SE–NW direction to be in the fault dip direction to delineate the subsurface features, especially Quaternary faults. Three seismic lines have been depicted, specifically Line_1, Line_2, and Line_3, and plotted on a Google Maps interface (Fig. 4). These lines are oriented in a southeast-to-northwest direction, aligned with the dip direction of the fault, with the aim of investigating the presence of quadruple faults. The red lines in the figure correspond to Quaternary faults that have been identified through geological analysis. The selection of the seismic Line_1 location was deliberate, strategically positioned between two historical earthquake events, as marked in the figure. These events occurred within the timeframe spanning from July 22, 2014, to July 18, 2014^13^.

## Geology and stratigraphy setting

The Gabal Ataqa area is placed in the northern part of the Eastern Desert of Egypt, about 120 km east of Cairo and 45 km south of Suez (Fig. 1). It is situated on the western side of the Gulf of Suez and is part of the Neoproterozoic basement complex of the Eastern Desert.

**Figure 5:**
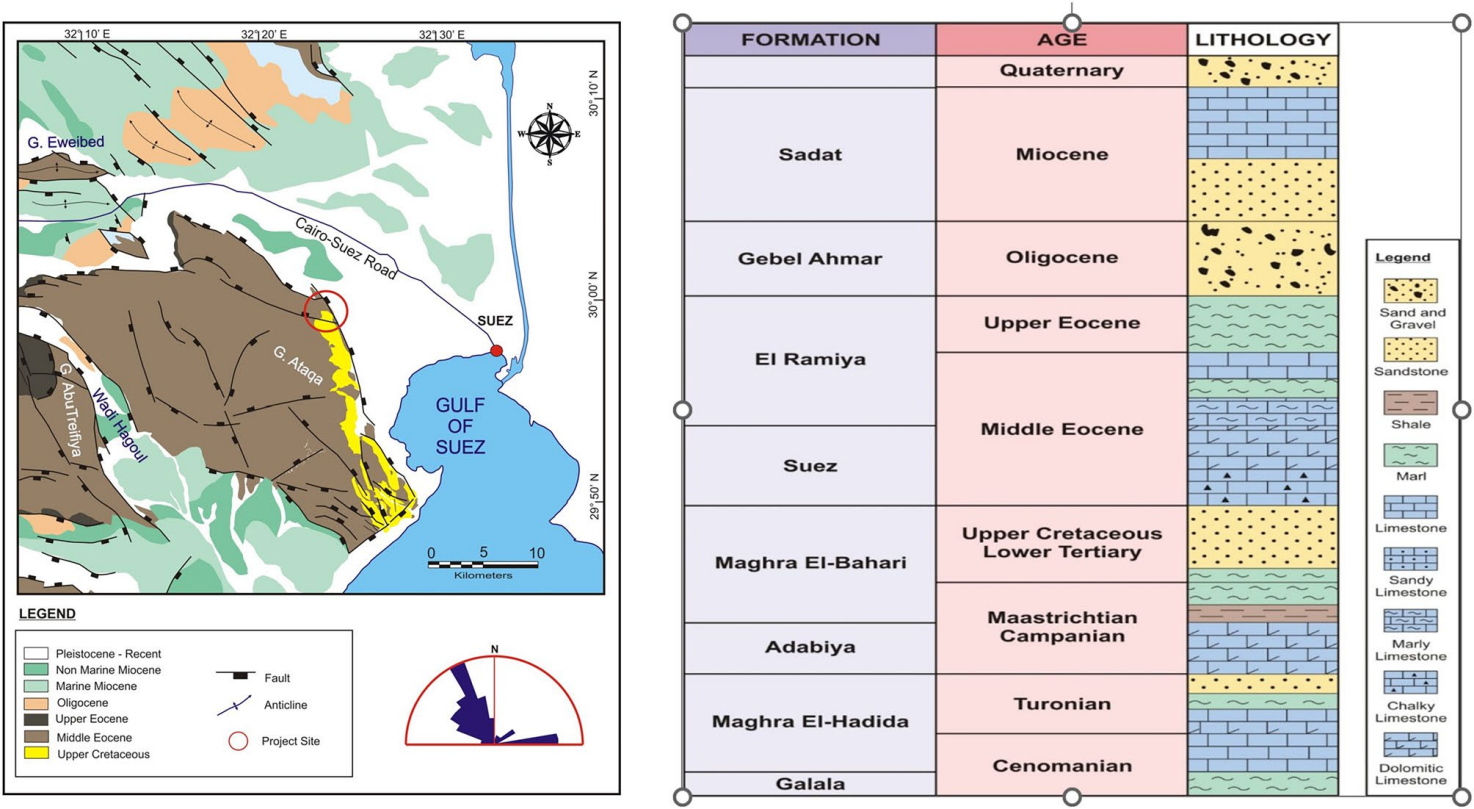
(**a**) Local geological map of the study area, the red circle in the map refers to an old geological project (21 Sep. 2020) located in the Gabal Attaqa area; (**b**) Generalized stratigraphic column of the exposed rock units in the Gabal Attaqa area.

The rocks in the Gabal Ataqa area are dominated by granitic and granodioritic plutons of the late Pan-African age, which intruded into metavolcanic and metasedimentary rocks of the older Bir Umq Formation. The Bir Umq Formation consists of metavolcanic rocks, such as basalt, andesite, rhyolite, and metasedimentary rocks, such as quartzite, phyllite, and schist. In addition, there are younger sedimentary rocks, such as sandstone, shale, and limestone, that overlie the basement rocks and form the northern part of the Gabal Ataqa area (Fig. 5a). The area is also known for its mineral deposits, such as gold, copper, and zinc, which are associated with granitic and granodioritic plutons^14–19^.

The stratigraphic column of the Gabal Ataqa area is well-documented (Fig. 5b). The geology of the area is dominated by a sequence of sedimentary rocks that record the geological history of the region over millions of years^20–24^. The stratigraphic column of the area includes many formations as illustrated in Table 1. It is worth noting that the stratigraphy of the Gabal Ataqa area is complex, with multiple sequences of sedimentary rocks stacked on top of each other in a series of thrust faults and folds. Accordingly, the actual sequence of rocks encountered in the area may vary depending on the location and depth of the drill hole or outcrop.

**Table 1:** The stratigraphic column of the study area in ascending order.

Formation	Composition
Top Nukhul Formation	Limestone, sandstone, and marl of Eocene age (56–34 million years ago). It is composed of alternating layers of limestone and marl, with sandstone beds towards the top
Rudeis Formation	Sandstone and shale of Eocene age. It is composed of interbedded sandstone and shale layers, with occasional limestone beds
Sudr Formation	Limestone and shale of Eocene age. It is composed of alternating layers of limestone and shale, with occasional sandstone beds
Matulla Formation	Sandstone and shale of Oligocene age (34–23 million years ago). It is composed of interbedded sandstone and shale layers
Gebel Ahmar Formation	Limestone and sandstone of Oligocene age. It is composed of alternating layers of limestone and sandstone
Ras Gharib Formation	Sandstone and shale of Miocene age (23–5 million years ago). It is composed of interbedded sandstone and shale layers
Kareem Formation	Limestone and sandstone of Miocene age. It is composed of alternating layers of limestone and sandstone
Belayim Formation	Sandstone and shale of Miocene age. It is composed of interbedded sandstone and shale layers
Gebel El Zeit Formation	Sandstone and shale of Miocene age. It is composed of interbedded sandstone and shale layers
Abu Zenima Formation	Sandstone and shale of Miocene age. It is composed of interbedded sandstone and shale layers
Bottom Nukhul Formation	Repeated in the stratigraphic column, with a younger sequence of limestone, sandstone, and marl overlaid on the older sequence

## Methodology and workflow

The methodology is based on the seismic reflection technique, including analysis of the acoustic waves reflecting from an acoustic interface. Acoustic interfaces can be formed from contacts between geological strata. These analyses can obtain a geologic cross-section after time-to-depth conversion and interpretation of geologic features. The common depth point (CDP) was applied to get the subsurface reflectors. The CDP method included time corrections of wavelet arrivals (based on absolute offset and velocity) to be adjusted as a vertical incidence, followed by combining all wavelets in the common depth point domain (CDP domain). The final result is a depth section that simulates a geologic cross-section^25^.

**Figure 6:**
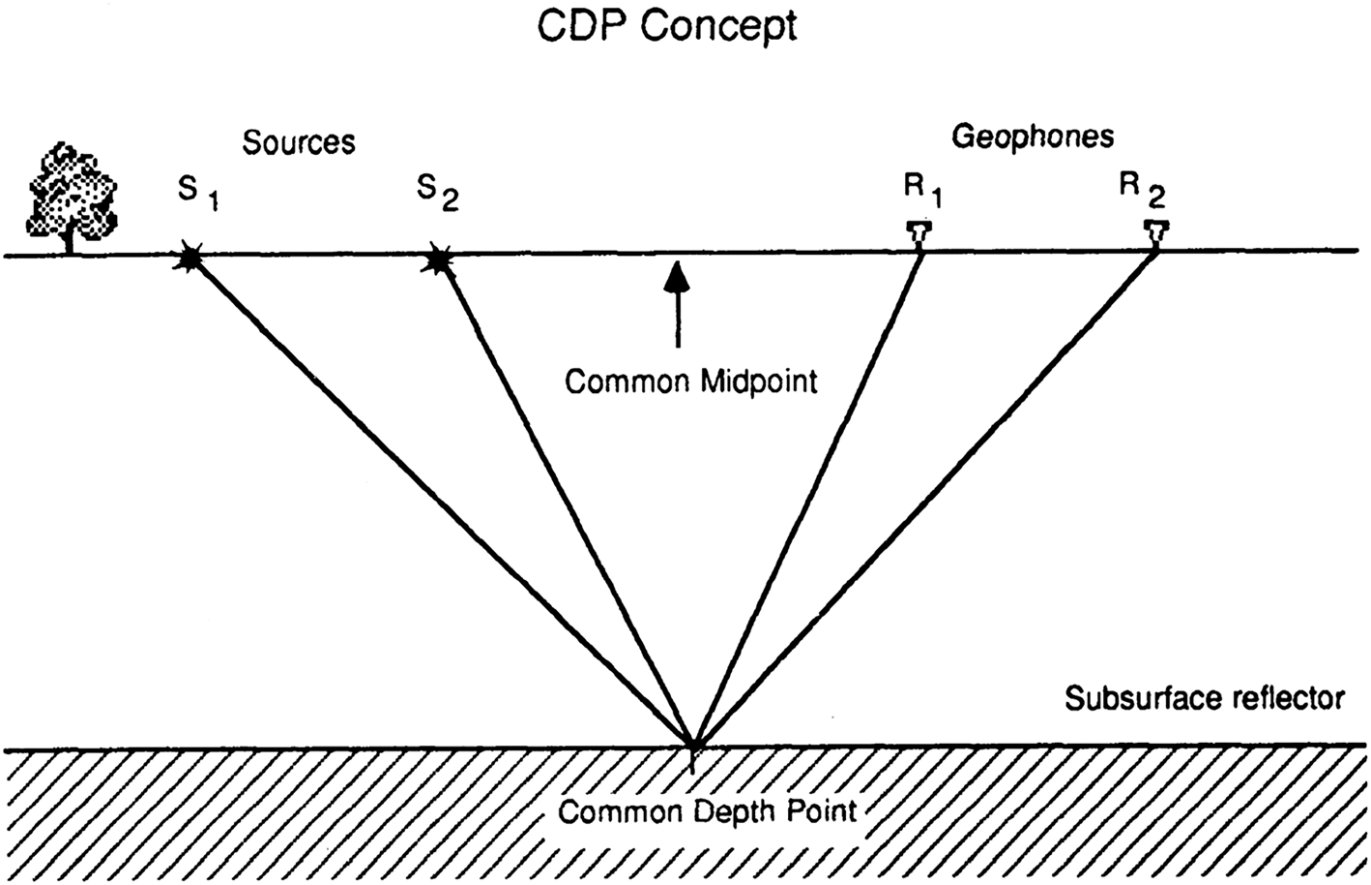
CDP Concept (Kansas Geological Survey).

An illustration of the CDP concept is represented in Fig. 6. When using a 48-geophone setup with a geophone spacing of 5 m and shot points positioned at each geophone sites, the subsurface reflection points will undergo 24 samplings, leading to 24-fold common-depth point (CDP) data after processing. The final CDP stack is obtained through two main stages, staking all traces for each CDP in one trace and finally stacking all CDPs in one stack called CDP stack.

The shallow seismic reflection application differs from the deep seismic reflection application concerning the seismic sources, the number of receivers, and the shooting pattern. For example, 12 to hundred receivers with a receiver interval of 1–5 m are often used for shallow seismic reflection, while 120 to thousands of receivers with a receiver interval of 10–25 m are often used for deep seismic reflection^26^. Sledgehammers and mechanical weight drops are used as seismic sources for shallow seismic reflection, while underground explosions and vibroseis are widely used for deep seismic reflection. In addition, the depth velocity model can be used to obtain a primary model for the shallow-surface layer^27^. The utilization of the shallow reflection technique encompasses three key phases: Acquiring data, processing the collected data, and interpreting the results.

### Field data acquisition

**Figure 7:**
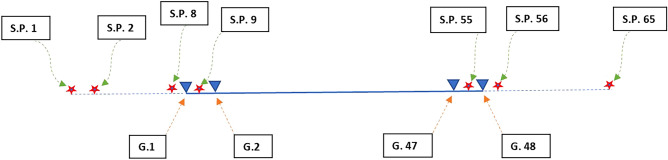
Shooting pattern (Abbreviations: S.P. is the shot point and G. is the Geophone).

The seismic data has been acquired using the Geometrics Strataview Acquisition System (Provider, Solgeo/Geometrics). The weight drop was used as a seismic source. The ground was well compacted, which helped in the non-distortion of the acoustic energy. Consequently, most of this energy has been penetrated to the shallow subsurface strata, reflected, and finally recorded as an SEG-Y format. Figure 7 shows the shooting pattern used in the current study. The acquisition parameters that have been used in this survey are represented in Table 2.

**Table 2:** Acquisition parameters.

Total number of shots	65
Shot point interval	5 m
Geophone interval	5 m
Sample rate	0.25 ms
Record length	500 ms
Number of vertical shot stack shots	5
Channels per record	48
Shooting pattern	Fixed-spread shooting pattern

**Figure 8:**
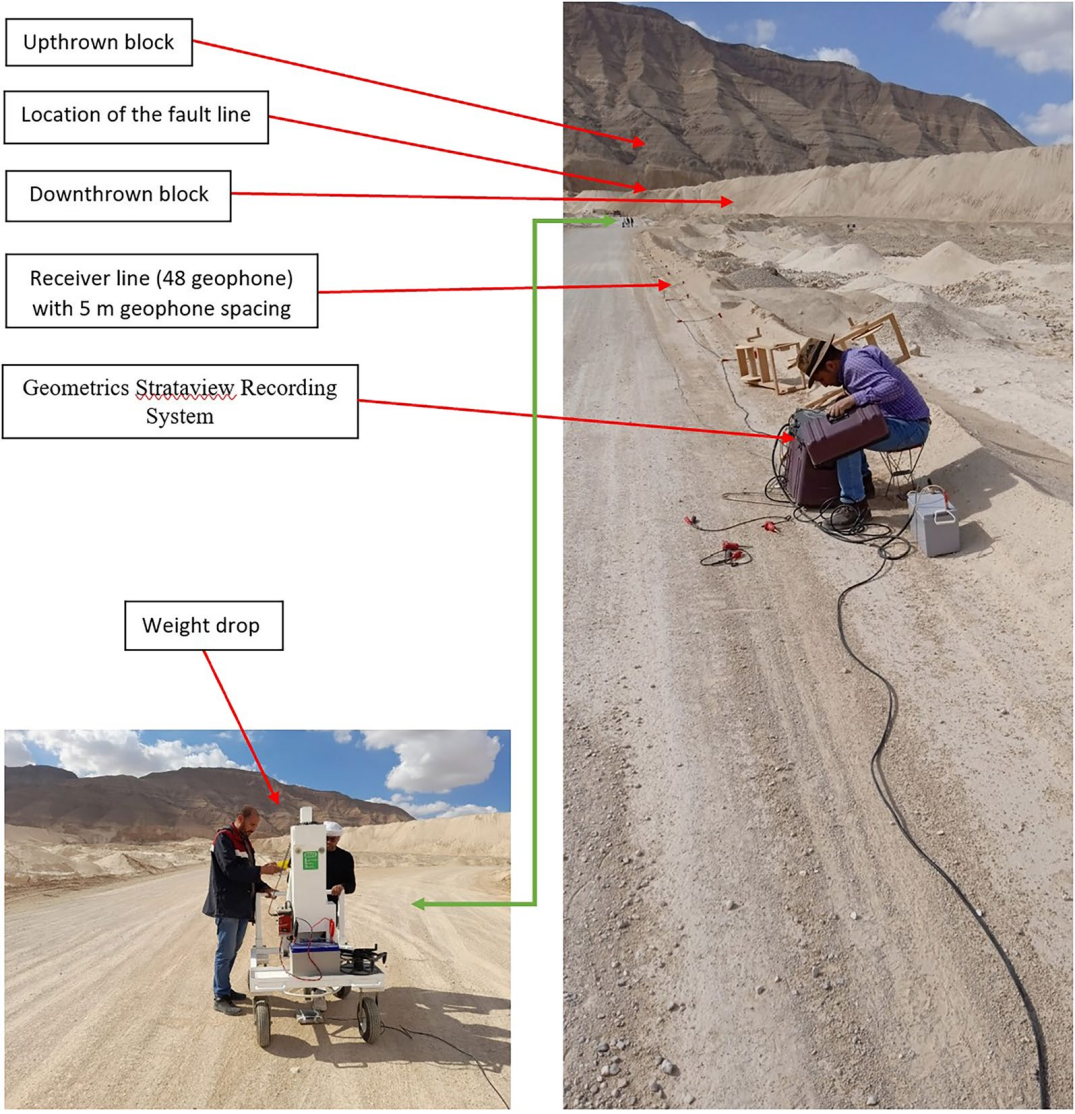
(**a**) Components of the acquisition system with an overview of the exposed Quaternary fault, (**b**) zoom in on the weight drop.

**Figure 9:**
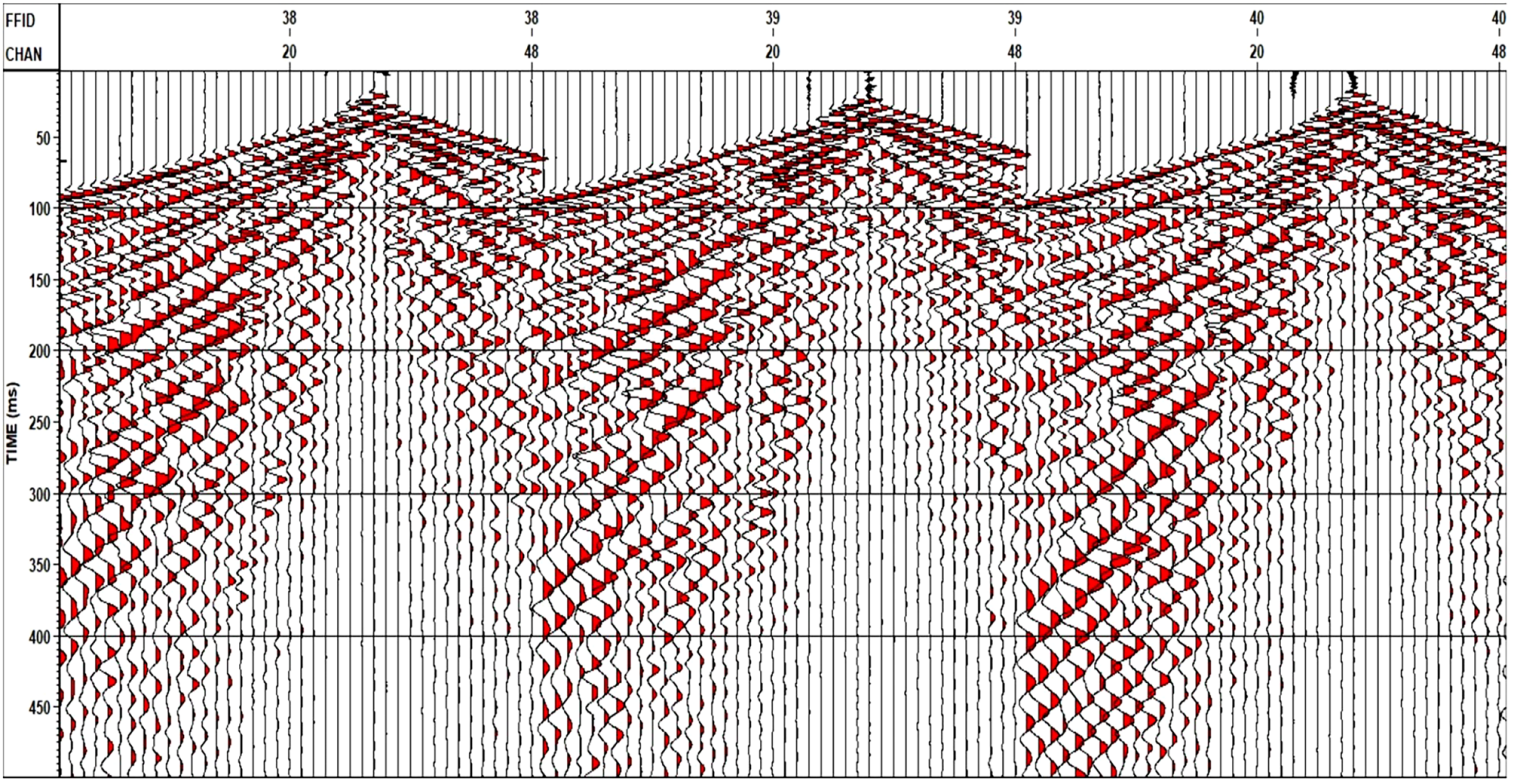
Shots example of raw data input.

The components of the acquisition system with an overview of an exposed quaternary fault (interpreted by the geological framework) are represented in Fig. 8. The three 2D seismic lines were acquired in the dip direction of this Quaternary fault. The field SEG-Y format has been converted to VISTA format to be a Raw Data Input for the processing sequence. Figure 9 represents shots example of the Raw Data Input.

### Seismic data processing

Conventional seismic data processing has been used to image the subsurface features. The standard and traditional process of analyzing seismic data usually consists of three primary stages: Deconvolution, stacking, and migration^28^. In addition, some processes should be applied, these processes include geometry, de-noise, static correction, and velocity analysis.

To correct the moveout effect in seismic data, the Normal Moveout velocity (NMO velocity) is estimated by finding the best match between the observed reflections and a hyperbolic approximation for each reflection time. This estimation is performed using the Common Depth Point (CDP) data collected during the process. The next Eq. (1) provides the reflection time when the medium above a flat reflector is uniform or homogeneous and exhibits isotropous properties. The elements of the equation are as shown in the illustrative Table 3. The time of reflection in Eq. (1) expresses a symmetrical or equal hyperbole.

1 $$ {\text{t}}\left( {\text{x}} \right)^{2} { } = {\text{t}}_{0}^{2} { } + {\text{ x}}^{2} /\nu^{2} $$

where (t) represents the travel time of seismic wave, (t_0_) denotes the two-way travel time to the zero-offset location, (x) represents the lateral offset from the zero-offset location, and (V) represents the velocity of seismic waves in the subsurface layer. However, for shallow large offset data, the hyperbolic approximation time fails, leading to a significant distortion in the seismic data with high frequencies generated by the velocity estimation and NMO correction^29^. In an effort to address these distortions, various research studies such as^30–33^, have explored the utilization of non-hyperbolic estimates to determine the time of reflection.

**Table 3:** Equation element symbol meaning of the Eq. (1).

Equation element symbol	Equation element symbol meaning
x	The distance between the source and receiver
t	The reflection time at position x = 0
v	The medium velocity
t (x)	The reflection time throughout the source-Reflector-receiver path

According to the previous explanation, a non-hyperbolic Normal Move Out has to be applied to the data to void the distortion frequently, especially in an anisotropic medium. The velocity analysis was applied every 7.5 m (every 3 CDPs) to get a better estimation for NMO velocity (stacking velocity). Vista 2d software has been used to process the data (Schlumberger software). A time and space Fourier transform in two dimensions (Fk) Post Stack Depth Migration and manual fault interpretation have been applied.

### Quality control (QC)

**Figure 10:**
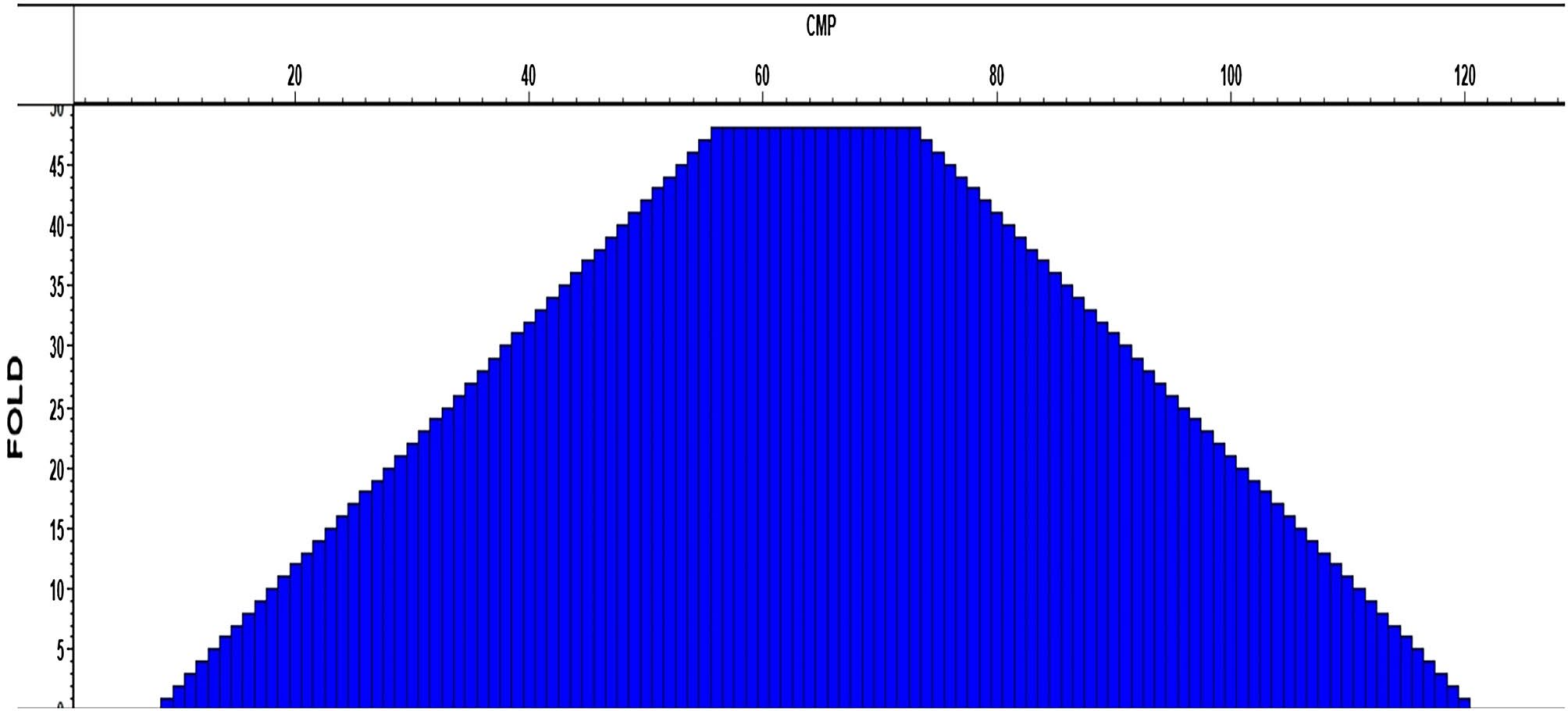
CDP fold Offset regularity QC.

Based on data QC, it was concluded that there were no problems with CDP fold, offset regularity or the CDP coordinates as shown in the following:

1. The Common Depth Point (CDP) fold can be calculated by Eq. (2), or by Eq. (3) as described in the Schlumberger energy glossary:
  2 $$CDP\,fold= \frac{\mathrm{The\, number\, of\, seismometer\, groups }}{2 *\mathrm{ The\, number\, of\, group\, intervals\, between\, shot\, points}}$$
  3 $$CDP\, fold= \frac{\mathrm{No}.\mathrm{ geophones }*\mathrm{ geophone\, spacing }}{2 *\mathrm{ shot\, spacing}}$$
2. By substituting the above equations with the survey parameters, CDP fold = 24. Figure 10 indicates that the dominant fold of the data is equal to 24.

**Figure 11:**
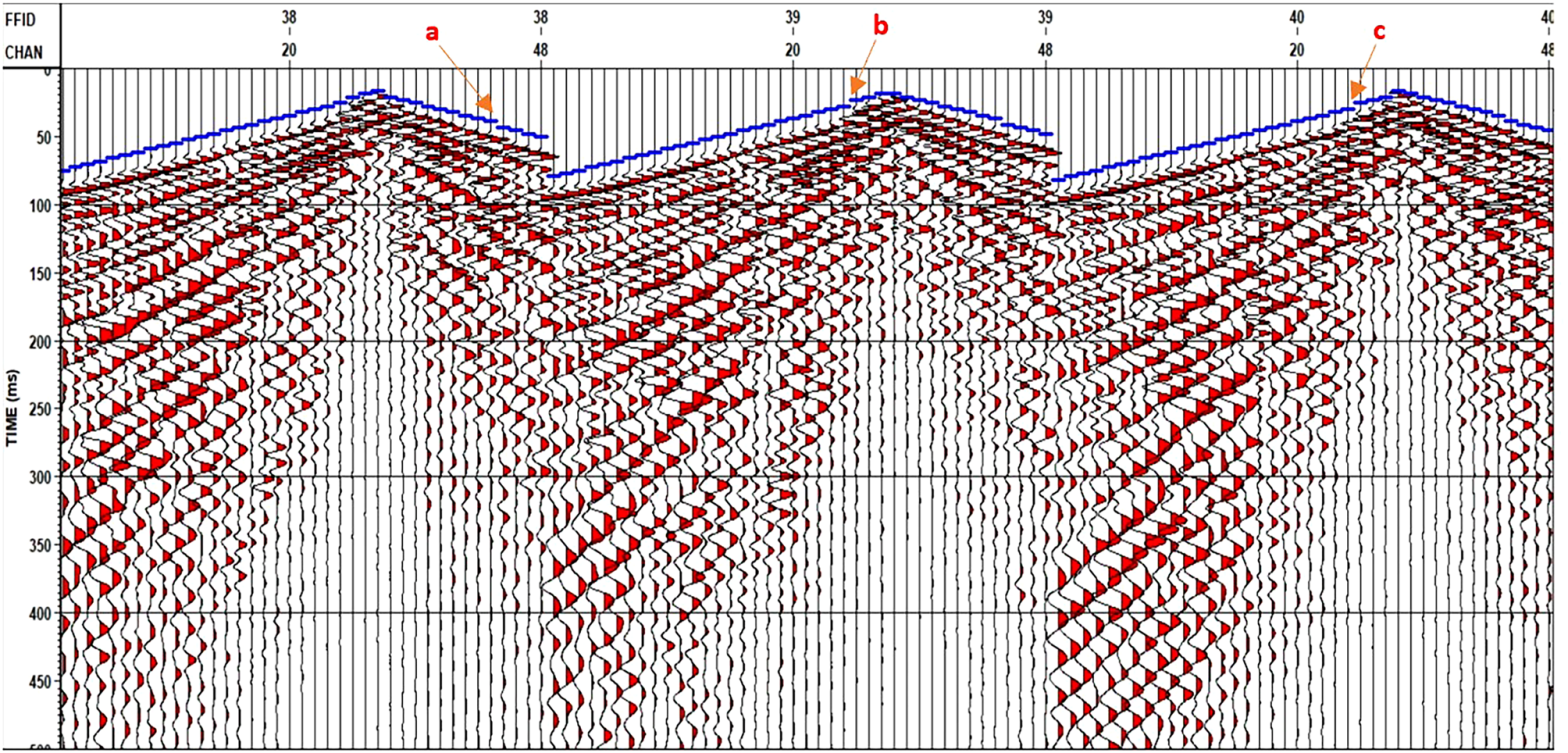
Offset QC (Positions a, b, and c are trace edit positions).

A top-mute QC based on the signed offset has been applied to the data to ensure that all offset values of a shot or receiver gather are regulated, as shown in Fig. 11.

**Figure 12:**
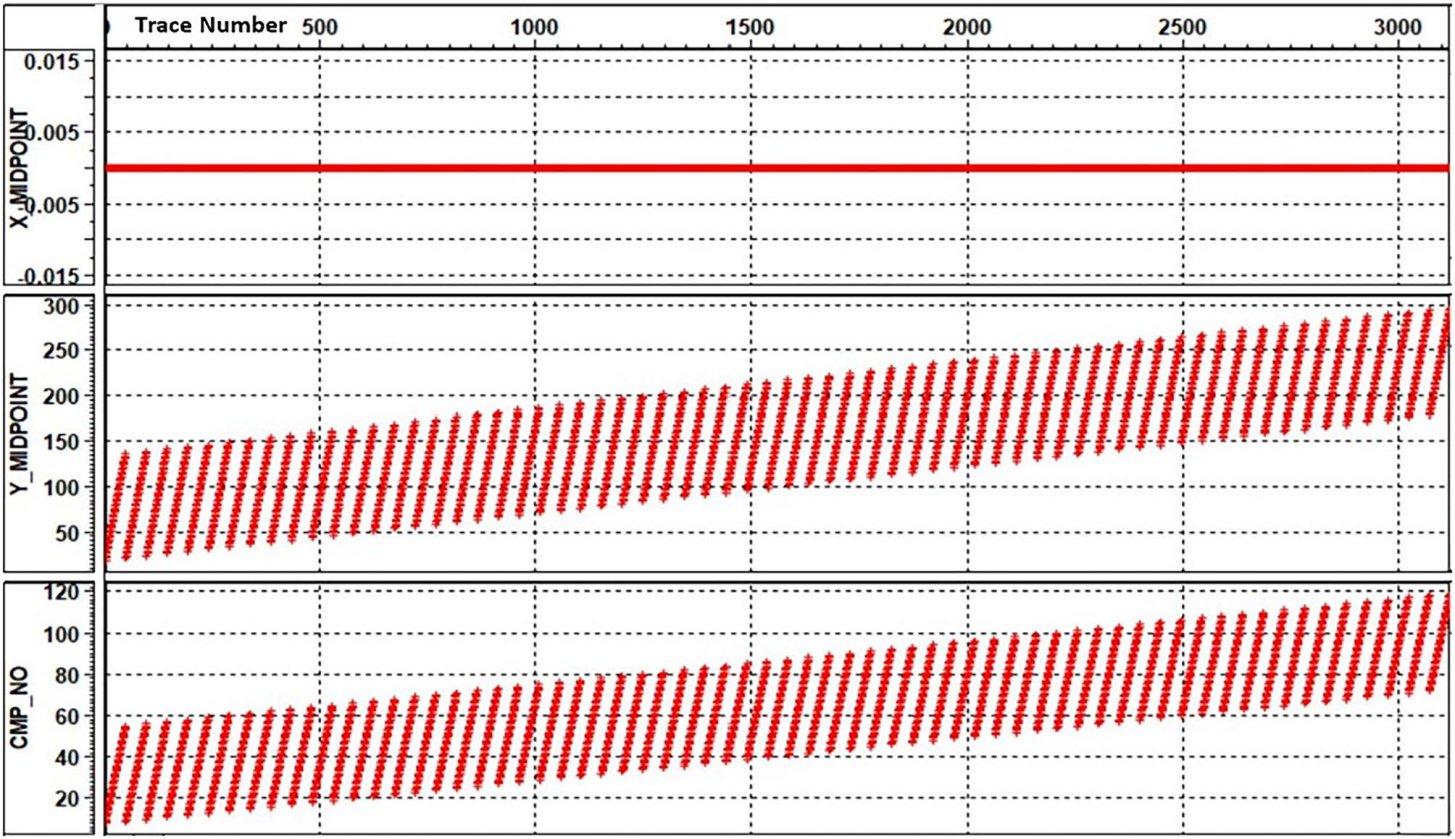
CDP coordinates QC.

**Figure 13:**
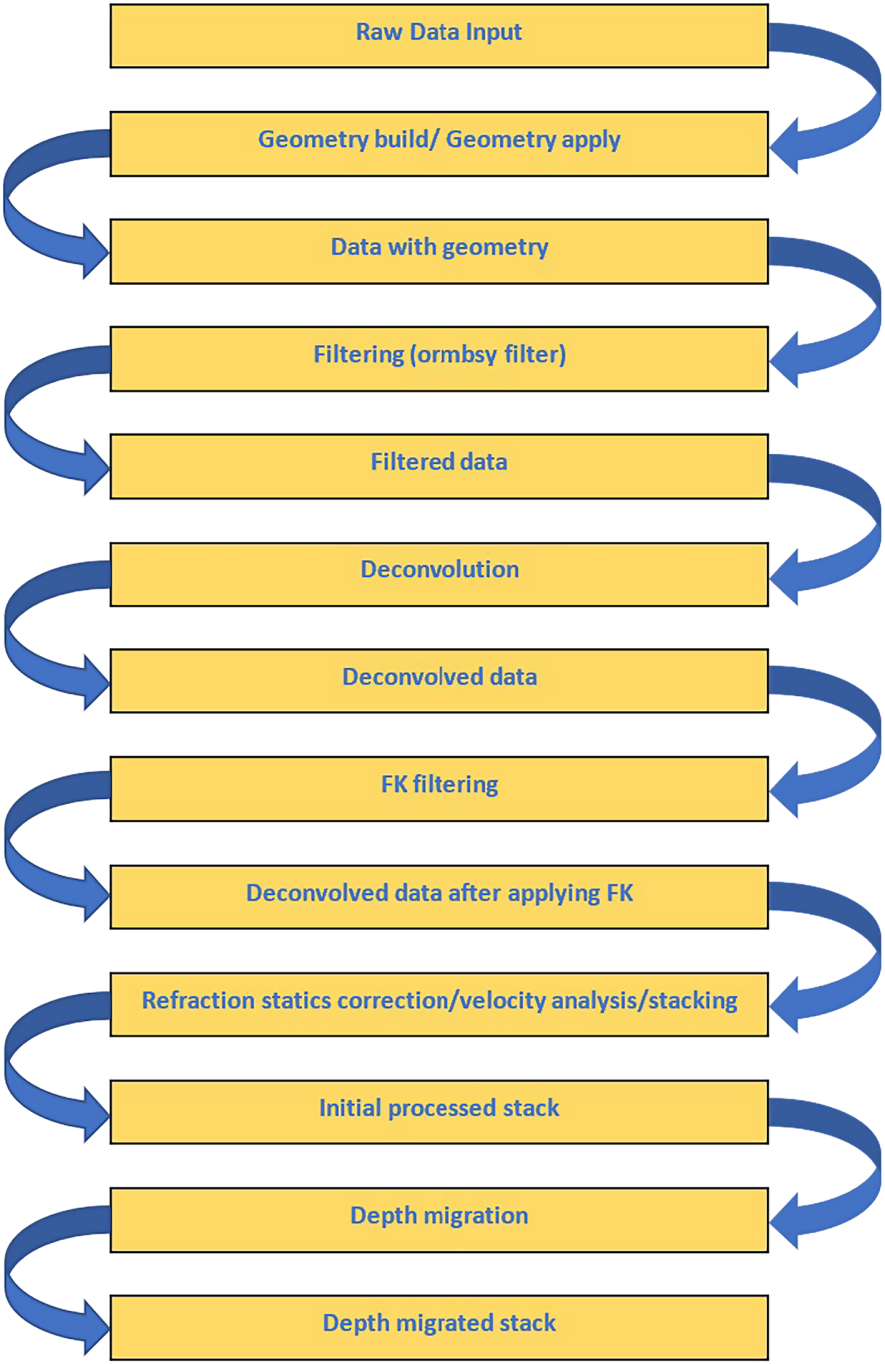
Processing sequence used in this study.

The QC on the CDP coordinates appear a clear regularity (Fig. 12), while the processing sequence that has been used in this study illustrated in Fig. 13. Through extensive testing, all processing parameters were identified. Table 4 displays the specific processing parameters that were used in this study.

**Table 4:** The specific processing parameters.

Deconvolution type	Predictive deconvolution
Deconvolution operator length	40 ms
Deconvolution predictive lag	20 ms
Migration technique	F–K migration
Normal moveout type (NMO type)	Non-hyperbolic normal move out

## Results

**Figure 14:**
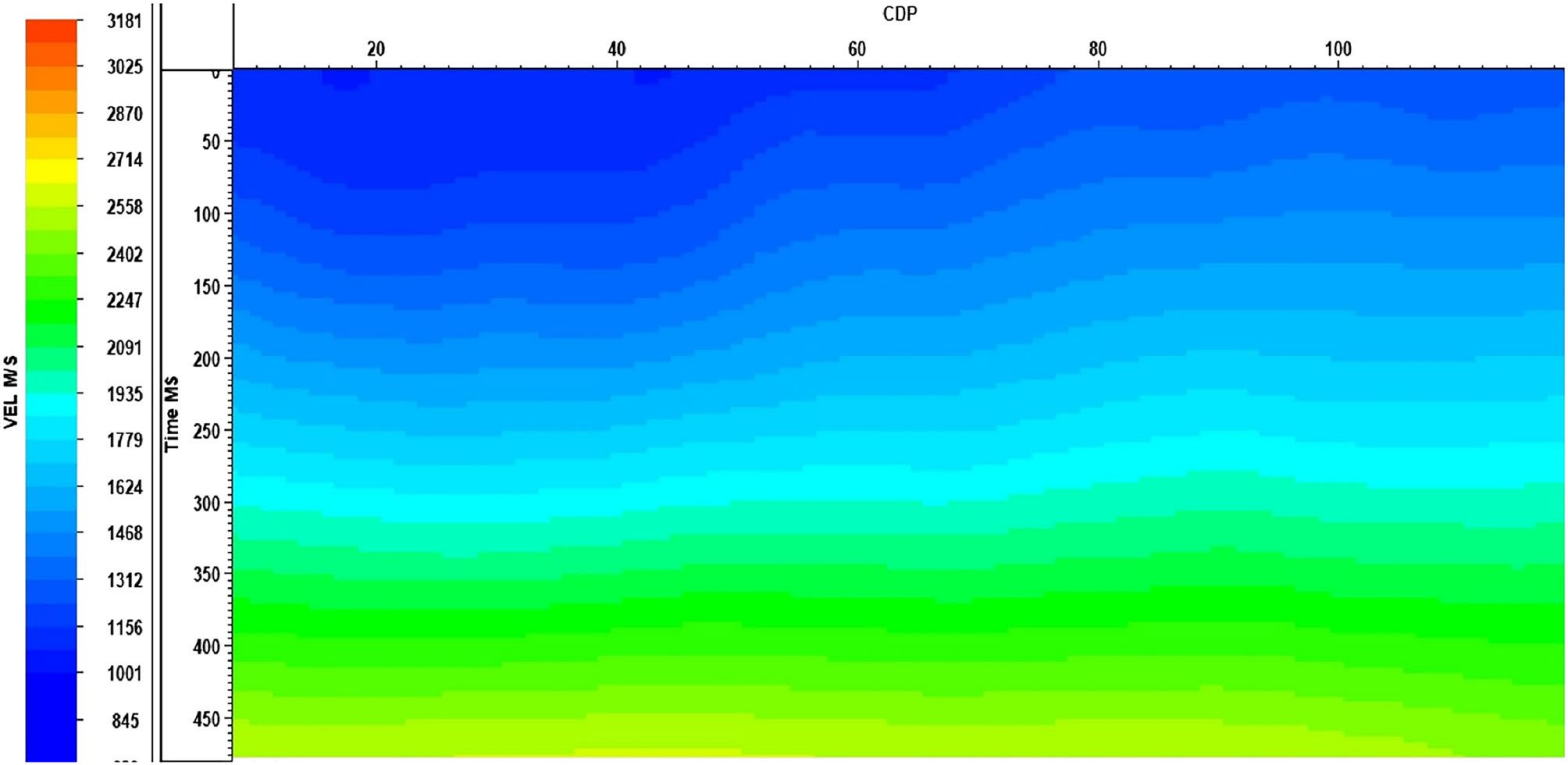
Graph Velocity View–Line 1.

Initial velocity values are hand-picked to drive the NMO velocity. A second pass of velocity work is applied to the deconvolution data. Finally, an update before migration was used to obtain the depth velocity model required to generate the final depth migration stack. Figure 14 illustrates the graph velocity view of Line 1 as an example of the study area velocity (after applying velocity analysis and the iso-velocity process).

**Figure 15:**
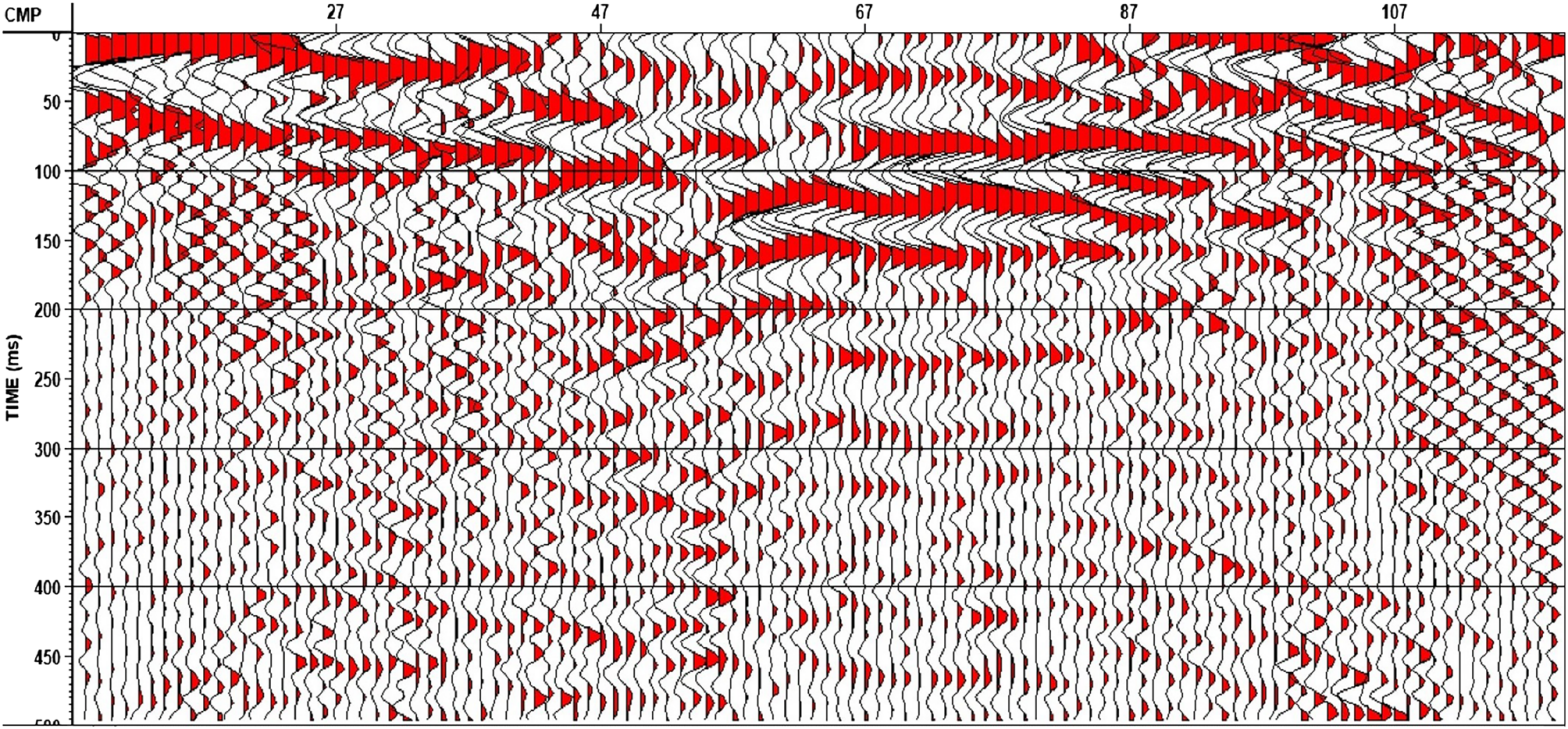
Final Time Processing Stack–Line 1.

Time processing included noise attenuation, deconvolution, and F–K filtering to enhance the ratio of the amount of signal to the amount of noise. Corrections were made for variations in the receiver and shot amplitudes caused by acquisition irregularities, followed by refraction statics correction. Figure 15 represents the final time processing stack for line 1.

**Figure 16:**
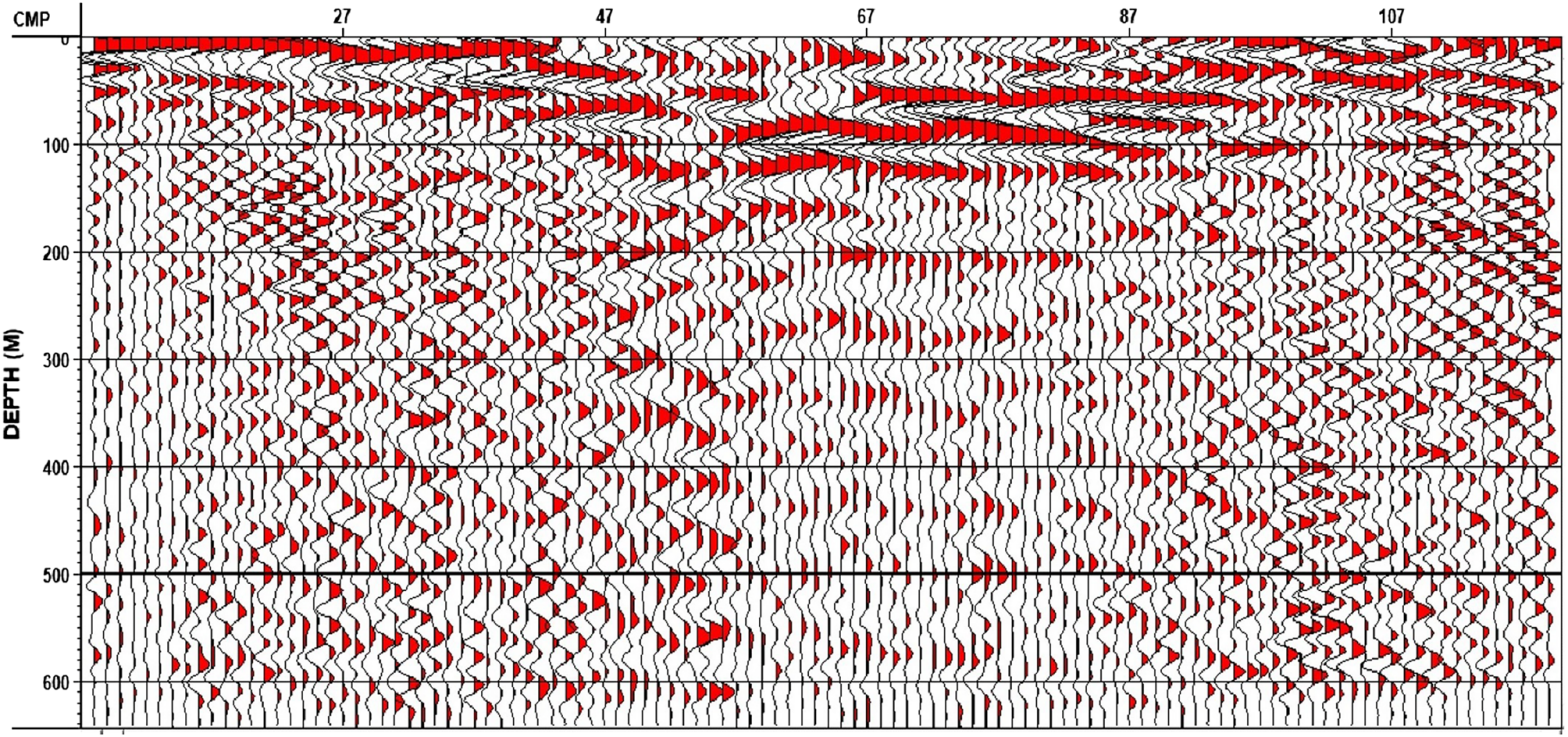
Final Depth Migration Stack–Line 1.

After applying comprehensive testing, the depth migration (FK migration) was applied using a base velocity of 1770 m/s and a CDP offset interval of 2.5 m. A set of improvement modules were used in the post-migration processing to enhance stack response. The image has significantly better fault resolution and event continuity. Figure 16 represents the final depth migration stack for Line 1.

**Figure 17:**
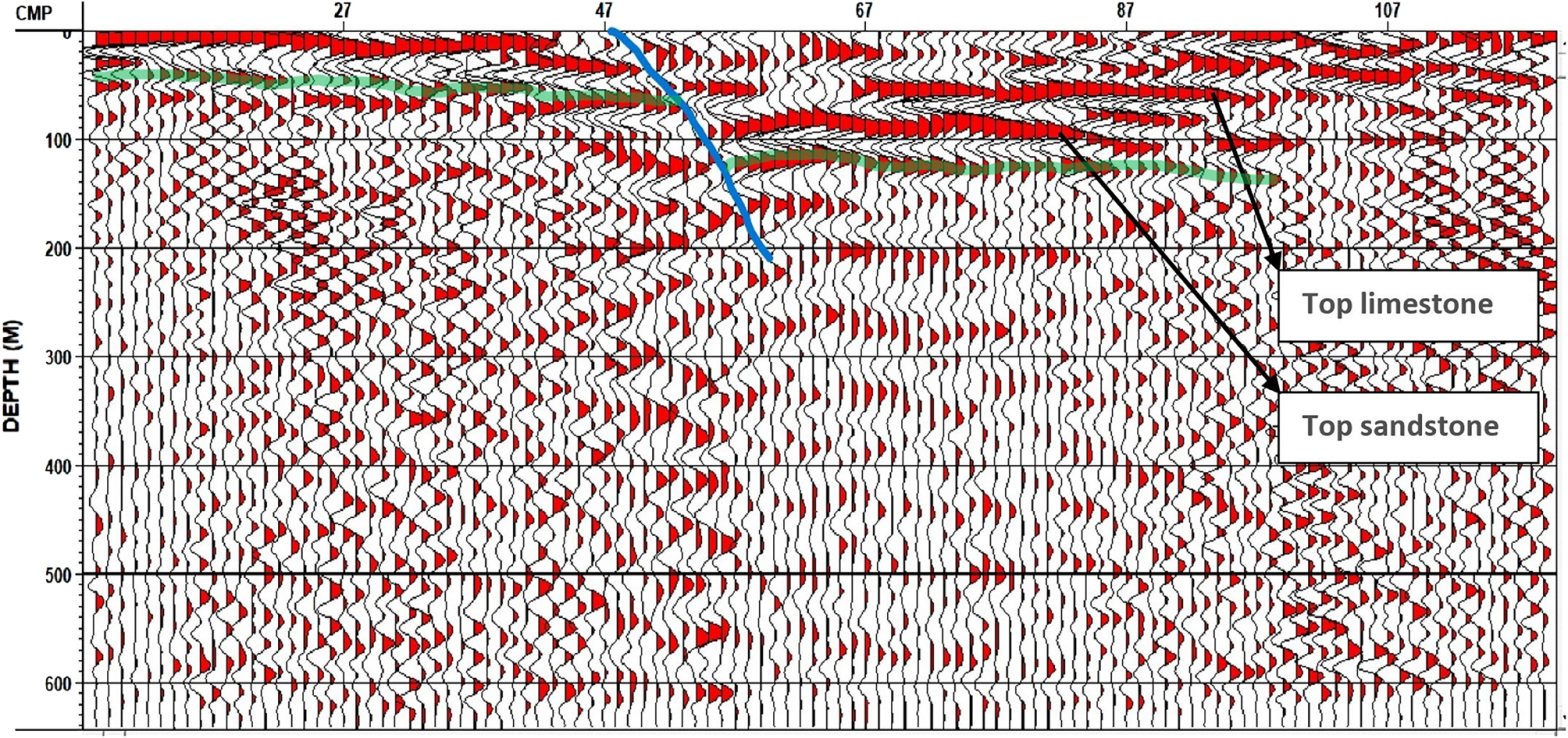
Final Depth Migration Stack with fault interpretation (blue line)–Line 1, (Lithology based on the generalized stratigraphic column of the exposed rock units in the Gabal Attaqa area in figure (5b)).

The Final Depth Migration Stack with fault interpretation for Line 1 indicates the presence of a quaternary fault. The upthrown block appears to be at the left side of the first line section, then the fault line, and finally, the downthrown block appears to be at the right side of the first line section (Fig. 17).

**Figure 18:**
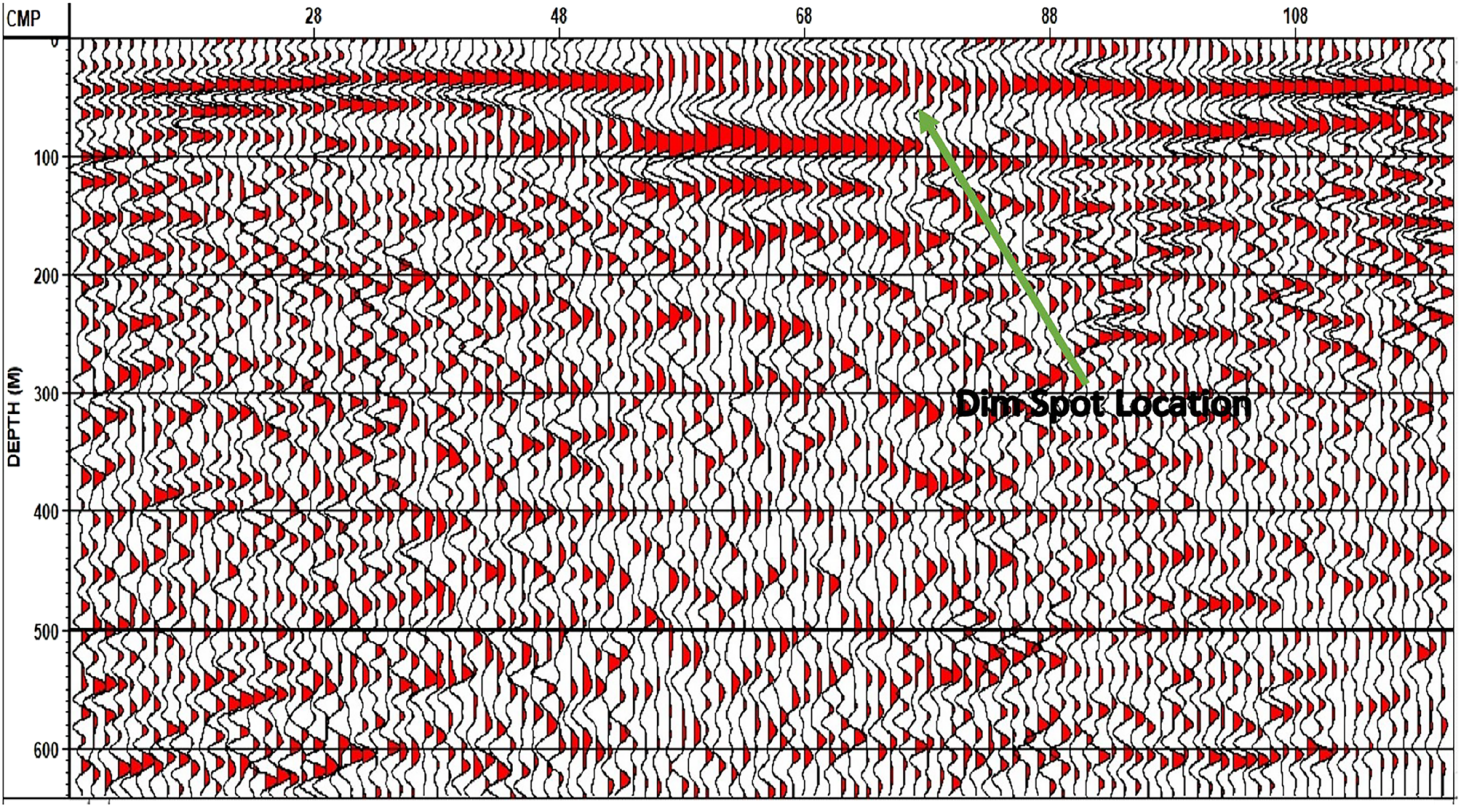
Final Depth Migration Stack for line 2 with dim spot indicator (Green arrow refers to dim spot location).

The final depth migration stack for line 2 indicates the presence of a dim spot. This dim spot has a local seismic characteristic characterized by a low amplitude anomaly at a depth of 50 m, which may indicate the presence of a groundwater aquifer or wet sandstone layer (Fig. 18).

**Figure 19:**
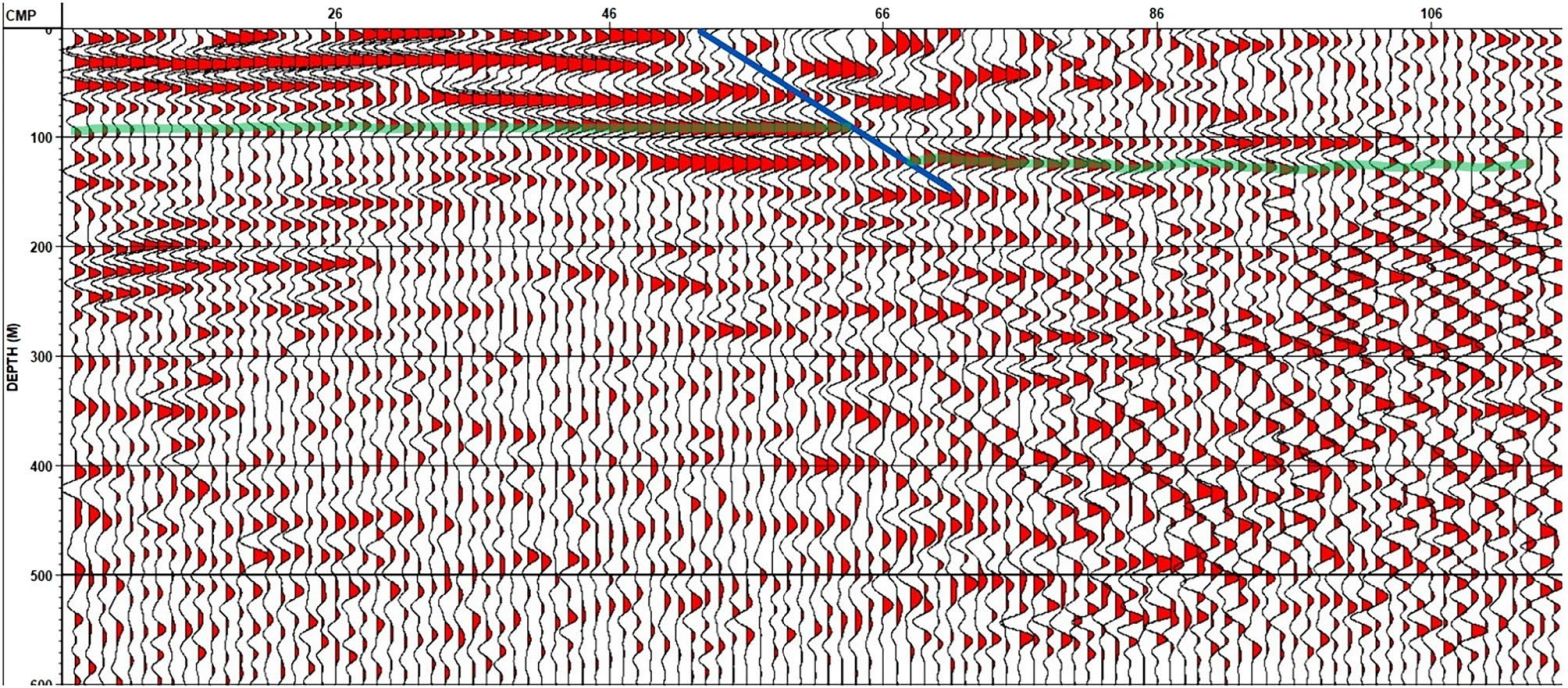
Final Depth Migration Stack with fault interpretation (blue line)–Line 3.

The final depth migration stack for line 3 indicates the presence of another Quaternary fault (Fig. 19). The two newly recognized quaternary faults are classified as normal faults, and their fault strike lines appear to be parallel to the strike line of the old recognized quaternary fault (a normal fault), which is interpreted by the geological framework. As a result, a step fault zone has been detected in the study area.

**Figure 20:**
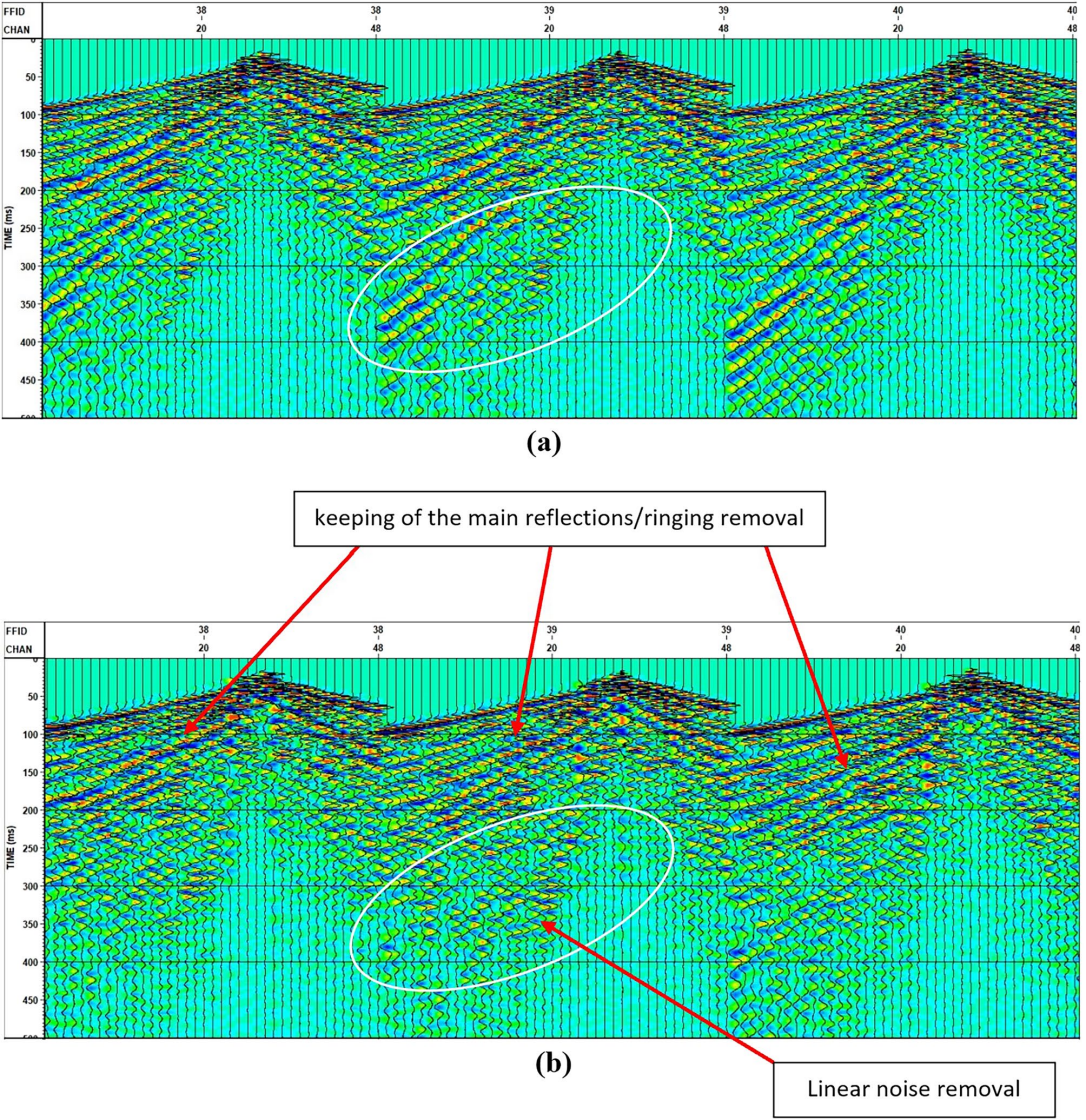
Shots (**a**) before and (**b**) after applying deconvolution and F-K filter.

**Figure 21:**
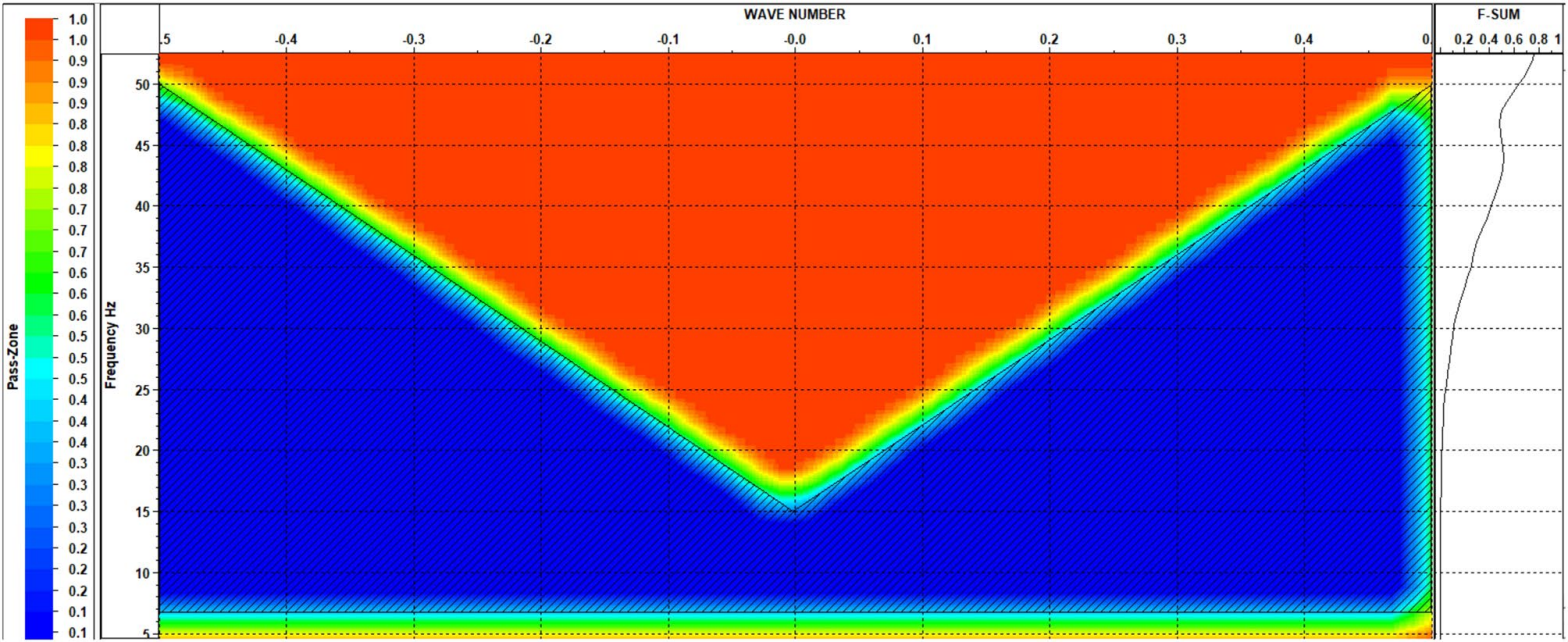
F-K polygon.

Deconvolution proves highly valuable in eliminating the influence of the source signature while preserving the representation of the earth’s response through its primary reflections. Additionally, it effectively mitigated most of the ringing. The application of F–K analysis is illustrated in Fig. 20 to eliminate linear noise, while Fig. 21 illustrates the F–K polygon that has been used.

**Figure 22:**
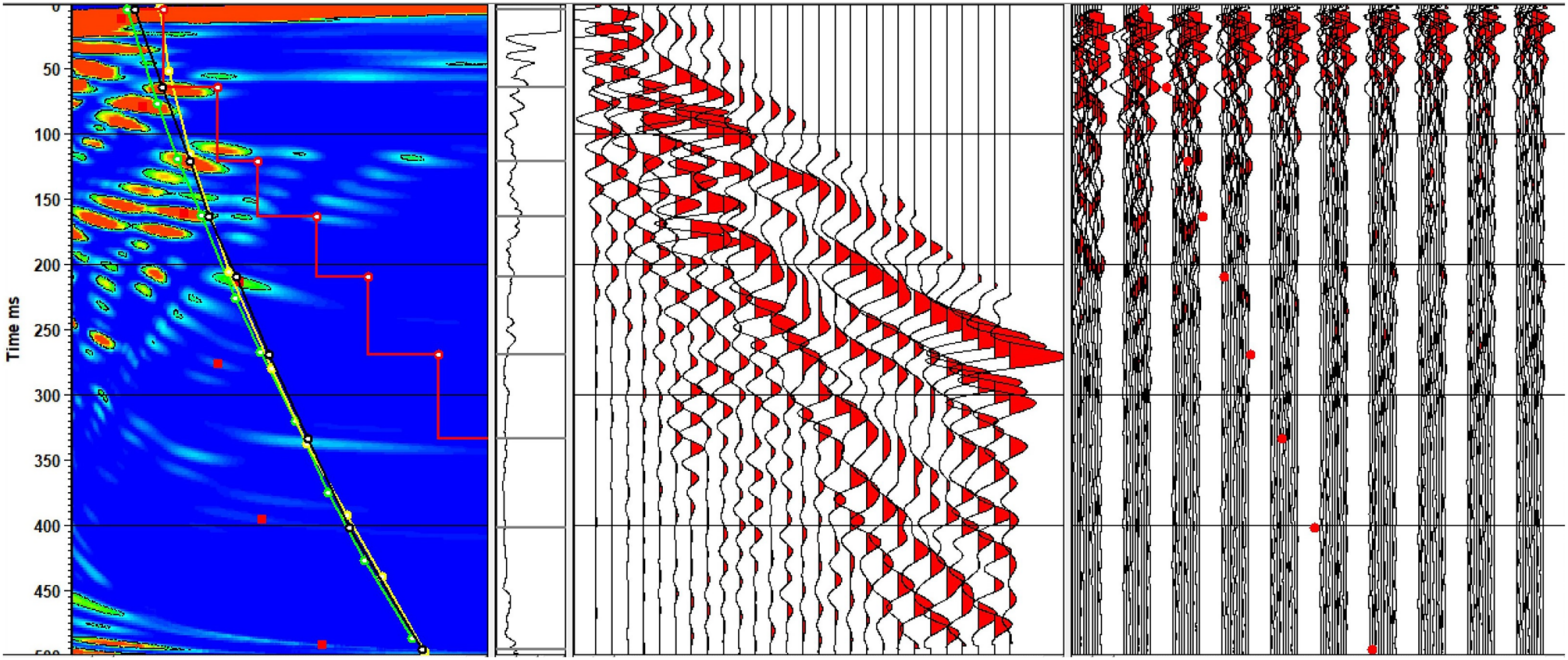
Velocity semblance combined with CDP gather and constant velocity stacks.

Clear diffractions, characterized by high-velocity values, and interbed multiples, associated with low-velocity values, have a notable impact on the velocity semblance. Avoiding these regions when determining the velocity trend significantly enhances the ultimate results. It is observed an instance of velocity semblance integrated with CDP gathers and constant velocity stacks (Fig. 22).

**Figure 23:**
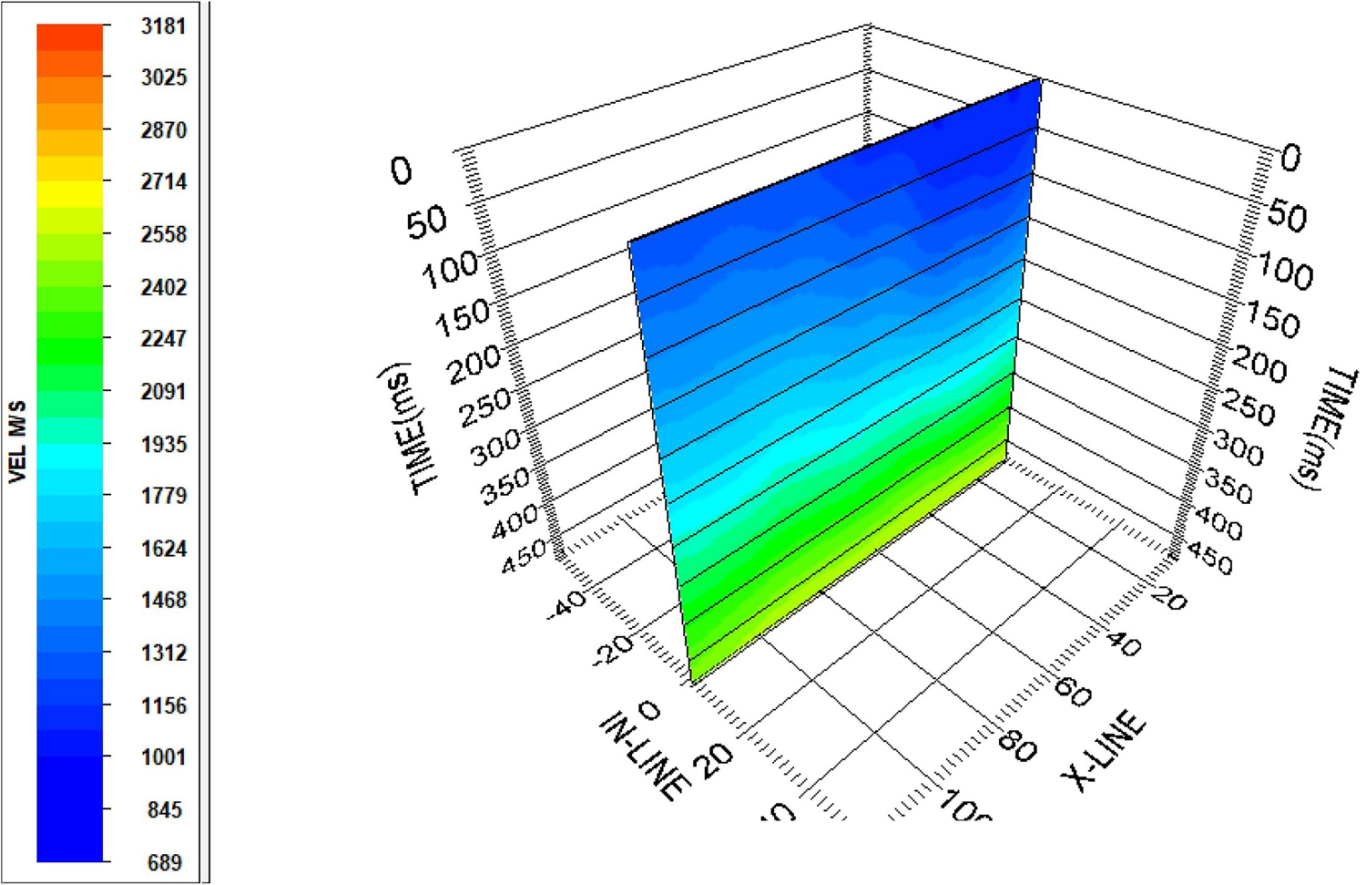
3D velocity view.

To provide a more comprehensive view of the velocity trend within the study area, a 3D velocity representation has been generated by introducing a dummy in-line number with CDP numbers corresponding to x-line numbers (Fig. 23).

**Figure 24:**
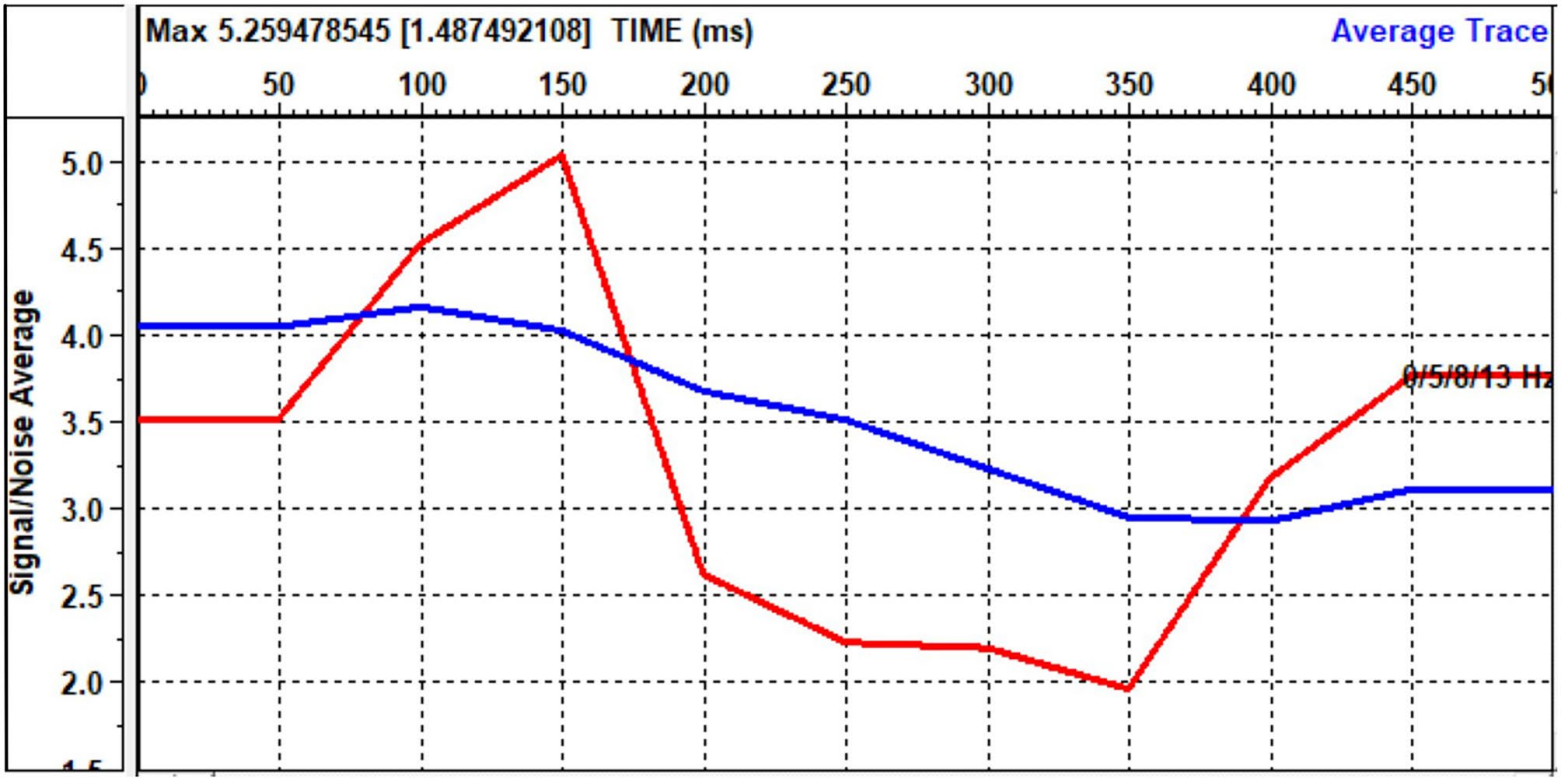
Signal-to-noise average.

Following the implementation of these preceding correction processes, the overall data quality has witnessed substantial improvements. VISTA software has computed an average signal-to-noise ratio based on all input data, which is then applied to refine the response of the final stacks. Figure 24 illustrates this average signal-to-noise ratio.

## Discussion

The initial data reveals the presence of two distinct types of noise that impact the signal-to-noise ratio: non-coherent noise and coherent noise. Manual trace editing effectively addressed the non-coherent noise, while some of it was also mitigated during the CDP stacking process. To tackle coherent noise, we employed the FK de-noise removal process.

This research showed that there are many steps that lead to the clarity of the measured data, but the most effective processes in improving the quality of the data are the deconvolution, F–K filtering, and Velocity analysis. Upon applying the predictive deconvolution technique, the results showcase the enhanced clarity of the primary reflections in the data, which were previously obscured by these two types of noise. The initial findings indicate evident lateral variations and significant high-frequency distortions. These distortions are successfully rectified by implementing velocity analysis at intervals of two and a half kilometers and employing non-hyperbolic approximations.

It's worth noting that the dips observed in the pre-migrated stacks do not appear to be genuine dips when compared to the dips in the depth-migrated stacks, which are more likely to represent the true dips for both subsurface strata and faults. Moreover, in the second seismic line, a distinct anomaly at a depth of 50 m raises the possibility of the presence of a groundwater aquifer or a saturated sandstone layer, possibly associated with sea transgression.

The findings presented in this study are subject to certain limitations and uncertainties such as:

1. The initial velocity values used for NMO velocity estimation were manually selected. This process introduces subjectivity and potential inaccuracies into the velocity model, which could impact the accuracy of depth migration results.
2. Multiple passes of velocity work, deconvolution, and other processing steps were applied to the seismic data. The variability in processing parameters and techniques can introduce uncertainties into the final results.
3. Refraction statics correction was performed to address variations in receiver and shot amplitudes due to acquisition irregularities, residual uncertainties may still exist, affecting the accuracy of the processed data.
4. The choice of parameters such as the base velocity (1770 m/s) and CDP offset interval (2.5 m) in depth migration can influence the interpretation of subsurface features. Variations in these parameters can lead to different outcomes.
5. The identification of geological features, such as faults and dim spots, is subject to interpretation. While efforts have been made to provide accurate interpretations, geological complexities and data limitations may introduce uncertainties and ambiguity.
6. While the VISTA software was used to improve the signal-to-noise ratio in the final stacks, it's important to note that some degree of noise reduction may not eliminate all noise components, potentially affecting the clarity of the results.
7. The effectiveness of the analysis is influenced by the density and distribution of seismic data points. Variations in data sampling density across the study area can lead to variations in the level of detail and accuracy achieved in different regions.

Despite these limitations and uncertainties, the presented findings offer valuable insights into the study area's subsurface structures and geological features. It's essential to consider these factors when interpreting the results and to recognize that further research and data refinement may lead to more precise assessments in the future.

## Conclusion

The main argument in the current study is that the shallow seismic reflection technique has proven to be a valuable method for analyzing underground structures. This argument is supported by several points. The use of the seismic reflection technique has resulted in enhanced and improved horizontal and vertical resolution within the study area. By adjusting parameters such as the receiver interval and the number of shots before and after the receiver line, the subsurface imaging quality has been significantly optimized and improved. The final stacked data exhibits notable improvements and enhances the data quality in event focusing, resolution, and noise reduction, suggesting the effectiveness of the technique. The study’s data processing and interpretation have led to the discovery and identification of two previously unknown Quaternary faults in the first and third seismic lines. The positioning and placement of the first seismic line between two historical earthquake events aim to increase the chances of pinpointing the sources of these earthquakes on the newly identified Quaternary fault. A potential subsurface anomaly in the form of a dim spot at 50 m depth in the second seismic line detected subsurface anomaly which suggested the presence of a water-wet sandstone layer or a groundwater aquifer.

Based on these findings, there is a strong warning and recommendation to relocate the industrial zone away from the three seismic lines, likely due to the identified faults and potential subsurface water-related features that could pose risks to development in these areas.

As a future recommendation suggested from the findings in this paper, it would be useful to enhance the current methodology used to detect subsurface structure in the study area, or in areas that exhibit similar conditions, using an additional geophysical technique. Possible methods to consider include electrical resistance tomography (ERT), magnetic surveys, gravimetric, or ground penetrating radar (GPR). This multi-modality approach can provide a more comprehensive, confirmatory, and robust understanding of subsurface structures.

## Data Availability

The data that support the findings of this study are available from [the Academy of Scientific Research and Technology (ASRT) in Egypt] but restrictions apply to the availability of these data, which were used under license for the current study, and so are not publicly available. However, the data will be made available upon reasonable request by addressing the fourth author (Mohammad Ezzelarb, mohezz@nriag.sci.eg) as the principal investigator (PI) for the project funded by the [Academy of Scientific Research and Technology (ASRT) in Egypt].
